# Enhancement by interleukin-1β of AMPA and NMDA receptor-mediated currents in adult rat spinal superficial dorsal horn neurons

**DOI:** 10.1186/1744-8069-9-16

**Published:** 2013-03-28

**Authors:** Tao Liu, Chang-Yu Jiang, Tsugumi Fujita, Shi-Wen Luo, Eiichi Kumamoto

**Affiliations:** 1 Center for Laboratory Medicine, the First Affiliated Hospital of Nanchang University, Nanchang 330006, China; 2 Department of Pediatrics, the First Affiliated Hospital of Nanchang University, Nanchang 330006, China; 3 Department of Physiology, Saga Medical School, 5-1-1 Nabeshima, Saga 849-8501, Japan

**Keywords:** IL-1β, IL-1ra, Glutamate receptor, Whole-cell patch-clamp, Spinal dorsal horn, Pain

## Abstract

**Background:**

Proinflammatory cytokine interleukin-1β (IL-1β) released from spinal microglia plays an important role in the maintenance of acute and chronic pain states. However, the cellular basis of this action remains poorly understood. Using whole-cell patch-clamp recordings, we examined the action of IL-1β on AMPA- and NMDA-receptor-mediated currents recorded from substantia gelatinosa (SG) neurons of adult rat spinal cord slices which are key sites for regulating nociceptive transmission from the periphery.

**Results:**

AMPA- and NMDA-induced currents were increased in peak amplitude by IL-1β in a manner different from each other in SG neurons. These facilitatory actions of IL-1β were abolished by IL-1 receptor (IL-1R) antagonist (IL-1ra), which by itself had no detectable effects on AMPA- and NMDA-induced currents. The AMPA- but not NMDA-induced current facilitated by IL-1β was recovered to control level 30 min after IL-1β washout and largely depressed in Na^+^-channel blocker tetrodotoxin-containing or nominally Ca^2+^-free Krebs solution. Minocycline, a microglia inhibitor, blocked the facilitatory effect of IL-1β on AMPA- but not NMDA-induced currents, where minocycline itself depressed NMDA- but had not any effects on AMPA-induced currents.

**Conclusions:**

IL-1β enhances AMPA and NMDA responses in SG neurons through IL-1R activation; the former but not latter action is reversible and due to an increase in neuronal activity in a manner dependent on extracellular Ca^2+^ and minocycline. It is suggested that AMPA and NMDA receptors are positively modulated by IL-1β in a manner different from each other; the former but not latter is mediated by a neurotransmitter released as a result of an increase in neuronal activity. Since IL-1β contributes to nociceptive behavior induced by peripheral nerve or tissue injury, the present findings also reveal an important cellular link between neuronal and glial cells in the spinal dorsal horn.

## Background

Interleukin-1β (IL-1β), a 17.5-kDa polypeptide proinflammatory cytokine, is believed to play an important role in modulating neuronal excitability in the peripheral and central nervous systems (PNS and CNS, respectively) in addition to its immunoregulatory effects [1-4]. Recently, IL-1β is of special interest as it is released under conditions associated with persistent pain including peripheral tissue injury (inflammatory pain) [5-8] and peripheral nerve injury (neuropathic pain) [9-12]. In animal models of neuropathic pain, IL-1β mRNA and protein are up-regulated immediately or as long as 35 days post-surgery [10,11,13], concomitantly with the development of hyperalgesia (an increase in sensitivity to noxious stimuli) and allodynia (a pain sensation in response to light-touch). Numerous reports have shown that IL-1β is an extremely potent mechanical and thermal hyperalgesic agent when injected into any peripheral tissues [14-20]. For example, IL-1β increases the heat-evoked release of calcitonin gene-related peptide (CGRP) from rat cutaneous nociceptors [17], sensitizes cultured dorsal root ganglion (DRG) neurons to noxious heat by potentiating heat-activated inward currents [21], and increases the discharge rate of DRG neurons in rats [22,23].

Accumulating evidence suggests that IL-1β is involved in pain sensation *via* not only peripheral but also central mechanisms such as the enhancement of nociceptive neuronal excitation in the CNS. This is in agreement with literatures suggesting that IL-1β produces hyperalgesia and allodynia when administered intracerebra-ventricularly or intrathecally [24-28]. Although the source of increased IL-1β in the CNS is not clear, spinal IL-1β may be produced by glial cells (e.g., microglia and astrocytes) in different chronic pain states [5,11,29,30]. Blocking the activation of spinal cord glial cell prevents or delays the development of persistent pain [31]. These findings strongly indicate that over-expression of IL-1β in the CNS may be involved in glia-related persistent pain. Besides, several studies demonstrated that IL-1β directly modulates neuronal activity. For example, IL-1β contributed to a membrane depolarization of paraventricular nucleus neurons [32] or a membrane hyperpolarization of hypothalamic neurons [33]. Furthermore, IL-1 receptor type I (IL-1RI) is localized in the superficial layers of the spinal dorsal horn, an area which plays a pivotal role in modulation of pain transmission [29]. Mice genetically-impared in IL-1 signaling (e.g., a deletion of IL-1RI or IL-1 receptor accessory protein and an over-expression of IL-1R antagonist (IL-1ra)) exhibited a reduction in the extent of thermal hyperalgesia and mechanical allodynia, compared to wild-type (WT) controls [34-36]. It is possible that the effect of IL-1β on pain transmission could be directly mediated by an interaction with IL-1RI in the superficial dorsal horn neuron.

The substantia gelatinosa (SG; lamina II of Rexed) of the spinal dorsal horn receives nociceptive information from the viscera, skin and other organs through fine myelinated Aδ and unmyelinated C primary-afferent fibers [37]. Nociceptive information is modulated by a variety of endogenous systems in the spinal dorsal horn and then transferred to the CNS. It has been shown that activity-dependent modulation of α-amino-3-hydroxy-5-methyl-4-isoxazole-4-propionic acid (AMPA) and *N*-methyl-D-aspartate (NMDA) receptors in SG neurons greatly contributes to persistent pain development [38]. Electrophysiological findings have provided evidence that exogenous acute application of IL-1β enhances AMPA- or NMDA-induced currents in SG neurons [39]. However, the underlying cellular mechanism remains unclear. Current evidence suggested that IL-1β is involved in not only acute but also chronic pain. It is reported that IL-1β facilitates inflammatory pain by enhancing phosphorylation of NMDA receptor NR1 subunit [29]. This observation strongly indicates that IL-1β may have long-term actions on glutamatergic synaptic transmission in SG neurons. Since AMPA and NMDA receptors were modulated in a manner distinct from each other [38], we hypothesized that the enhancements of AMPA- and NMDA-induced currents produced by IL-1β in SG neurons are IL-1RI dependent while being different in cellular mechanism. We also thought that the IL-1β effects may be influenced by inhibition of glial activation. Spinal cord slice preparations show preserved local neuronal networks and thus have proved to be useful in analyzing the mechanisms of pain transmission. The aim of the present study is to provide additional evidence for the cellular mechanism of the effects of IL-1β on the AMPA and NMDA responses in SG neurons by using blind whole-cell patch-clamp recordings from adult rat spinal cord slices.

## Results

Whole-cell patch-clamp recordings were made from a total of 82 SG neurons. Stable recordings could be obtained from slices maintained for more than 12 h after the dissection, and recordings were made from single SG neurons for up to 4 h. All SG neurons tested had resting membrane potentials more negative than −50 mV (when measured in the current-clamp mode).

### AMPA- and NMDA-induced currents are enhanced by IL-1β in SG neurons

**Figure 1:**
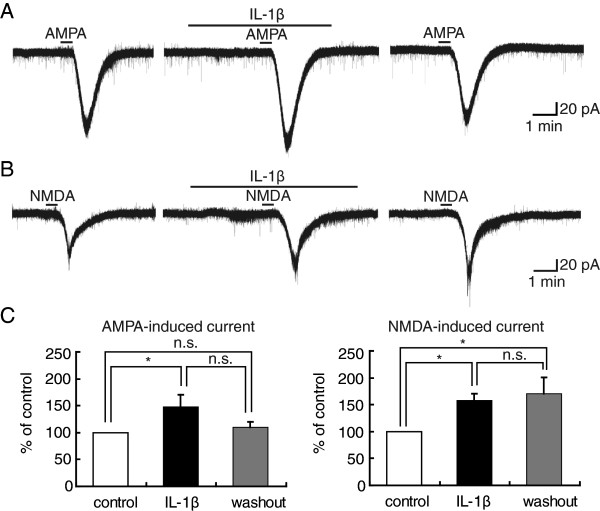
**Enhancement by IL-1β of AMPA- and NMDA-induced currents in rat substantia gelatinosa (SG) neurons.** (**A**) Recordings of AMPA-induced inward currents, which were enhanced in the presence of IL-1β and recovered at 30 min after its washout. Drug application is marked with bars. (**B**) Recordings of NMDA-induced inward currents, which were enhanced by IL-1β; this enhancement persisted at 30 min after its washout. (**C**) Bar graphs showing the averaged peak amplitude of AMPA (left, n = 12)- and NMDA (right, n = 7)-induced currents under the treatment with IL-1β (black bar) and after its washout (gray bar), relative to those before this treatment (control, open bar). In this and subsequent figures, vertical lines accompanied by bars indicate SEM; *: P < 0.05, compared with control; n.s.: not significant (*t* test). In this and subsequent figures, the concentrations of IL-1β used was 10 ng/ml; the concentrations of AMPA and NMDA used were 5 and 50 μM, respectively; the AMPA and NMDA responses were induced at holding potentials (HPs) of −70 and −50 mV, respectively.

Exogenous application of AMPA (5 μM) for 30 s induced an inward current at a holding potential of −70 mV. After 3 min pretreatment of IL-1β (10 ng/ml, in this and subsequent experiments, a total of its superfusion time depended on when the agonist-induced current returned to original level, usually 5–7 min), the peak amplitude of AMPA-induced current increased to 148 ± 22% (n = 12, P < 0.05) of control (139 ± 27 pA, Figure 1A), and returned to 110 ± 10% (n = 9, P > 0.05) of initial values (139 ± 36 pA) after IL-1β was washed out for 30 min (Figure 1A). Bath-applied NMDA (50 μM) for 30 s elicited an inward current at a holding potential of −50 mV (since opening of NMDA receptor-channel requires both its agonist and depolarization to relieve its block by Mg^2+^ for its activation). In the presence of IL-1β, the mean amplitude of NMDA-induced current enhanced to 158 ± 13% (n = 7, P < 0.05) of control (49 ± 12 pA, Figure 1B). These values were further increased to 170 ± 31% (n = 4, P < 0.05) of initial values (45 ± 12 pA) 30 min later after washout of IL-1β (Figure 1B), suggesting an irreversible effect of IL-1β on NMDA receptors. This indicates that the facilitatory effect of IL-1β on NMDA-induced currents lasts for at least 30 min even in the absence of this cytokine. Therefore, mechanisms underlying the facilitatory effects of IL-1β on postsynaptic AMPA and NMDA receptors may be different from each other. The effects of IL-1β on AMPA- and NMDA-induced currents were summarized in Figure 1C, which were examined in 12 and 7 neurons, respectively. In accordance with previous results [39], a part of SG neurons examined exhibited a IL-1β-mediated potentiation of AMPA- (12 out of 19 neurons, 63%) and NMDA-induced (7 out of 8 neurons, 88%) currents.

### The enhancement by IL-1β of AMPA- and NMDA-induced currents is IL-1 receptor-dependent

**Figure 2:**
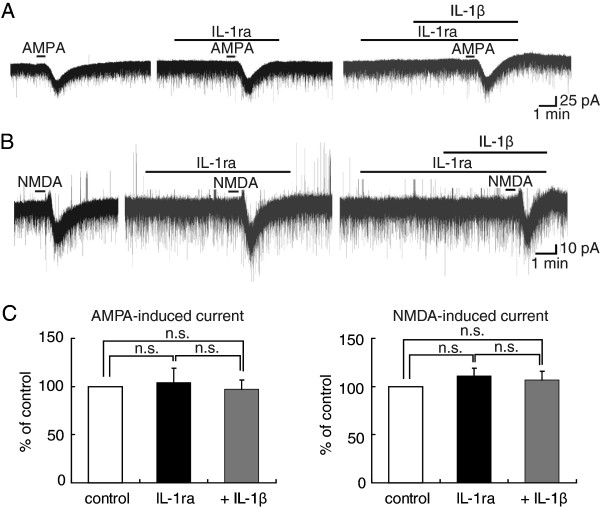
**AMPA- and NMDA-induced current amplitude increase produced by IL-1β was reduced in extent by IR-1ra.** (**A**, **B**) Recordings of AMPA (**A**) or NMDA (**B**)-induced currents in Krebs solution without drugs (left), with IL-1ra (50 ng/ml) alone (middle) and both IL-1ra and IL-1β (right); they were obtained from the same neuron. In each of (**A**) and (**B**), all recordings were obtained from the same neuron. (**C**) Peak amplitude of AMPA (left, n = 8)- and NMDA (right, n = 9)-induced currents in Krebs solution containing IL-1ra only (black bar) and together with IL-1β (gray bar), relative to those without IL-1ra and IL-1β (control, open bar).

IL-1β exerts its actions by binding to IL-1RI. To assess whether the effects of IL-1β on AMPA- and NMDA-induced currents in SG neurons were mediated by IL-1RI, we examined the actions of IL-1β in the presence of recombinant human IL-1ra. In these experiments, IL-1ra (50 ng/ml) was applied 3 min before treatment with IL-1β, and the drug remained present while the agonist was being applied and the subsequent stimulation with AMPA or NMDA. IL-1ra *per se* had no significant effects on AMPA- or NMDA-induced currents, and the amplitudes of the currents were, respectively, 104 ± 15% (n = 8, P > 0.05) of control (115 ± 40 pA, Figure 2A) and 111 ± 8% (n = 9, P > 0.05) of control (51 ± 16 pA, Figure 2B). Moreover, IL-1ra blocked the responses of IL-1β in all the neurons tested (Figure 2A, B). Amplitudes of the inward currents elicited by exogenous AMPA and NMDA applied in the presence of IL-1β in Krebs solution containing IL-1ra were, respectively, 97 ± 10% (n = 5, P > 0.05) of control (120 ± 26 pA, Figure 2A) and 107 ± 9% (n = 4, P > 0.05) of control (60 ± 20 pA, Figure 2B). Because excitatory transmission is mainly mediated by AMPA and NMDA receptors in SG neurons, these results indicate that the excitatory effects of IL-1β, which are significantly blocked by IL-1ra, are due to a direct activation of IL-1RI. Figure 2C summarizes the effects of IL-1β on AMPA- and NMDA-induced currents in the presence of IL-1ra, which are examined in 8 and 9 neurons, respectively.

### Characterization of the IL-1β effect on AMPA- and NMDA-induced currents

**Figure 3:**
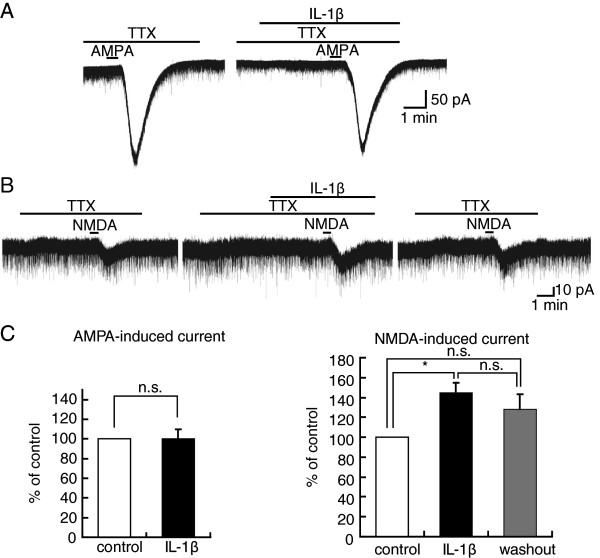
**Tetrodotoxin (TTX) reduced the AMPA- but not NMDA-induced current amplitude increase produced by IL-1β.** (**A**) Recordings of AMPA-induced currents in the absence and presence of IL-1β in Krebs solution containing TTX (0.5 μM); they were obtained from the same neuron. (**B**) Recordings of NMDA-induced currents in the absence and presence of IL-1β in Krebs solution containing TTX (0.5 μM); they were obtained from the same neuron. (**C**) The averaged peak amplitude of AMPA (left, n = 7)- and NMDA (right, n = 7)-induced currents in the presence of IL-1β (black bar), relative to those before the application of IL-1β (control, open bar), in Krebs solution containing TTX.

Since the function of postsynaptic AMPA and NMDA receptors can also be affected by any neuromodulators released as a result of neuronal activities, we examined the involvement of voltage-gated Na^+^-channel activation in the AMPA- and NMDA-induced current amplitude increase produced by IL-1β. Figures 3A and B demonstrate the effects of IL-1β on AMPA- and NMDA-induced currents, respectively, in Krebs solution containing a Na^+^-channel blocker tetrodotoxin (TTX; 0.5 μM). IL-1β did not affect AMPA-induced current under a pretreatment with TTX (100 ± 10% (n = 7, P > 0.05) of control (122 ± 37 pA), Figure 3A). In contrast, even in the presence of TTX, neurons showed a significant increase in NMDA-induced current amplitude after IL-1β treatment (145 ± 10% (n = 7, P < 0.05) of control (48 ± 8 pA), Figure 3B). Unlike the effect of IL-1β on NMDA-induced current in the absence of TTX (Figure 1B), IL-1β modulation of the NMDA response was moderately increased after its removal in the presence of TTX (128 ± 15% (n = 5, P > 0.05) of control (69 ± 12 pA), Figure 3B, C). Hence, TTX prevented the facilitatory effect of IL-1β on AMPA-induced currents while having no effect on NMDA ones. Figure 3C summarizes the effects of IL-1β on AMPA- and NMDA-induced currents in the presence of TTX, each of which effects is examined in 7 neurons. These results suggest that the potentiation of AMPA but not NMDA current is due to an increase in neuronal activity.

**Figure 4:**
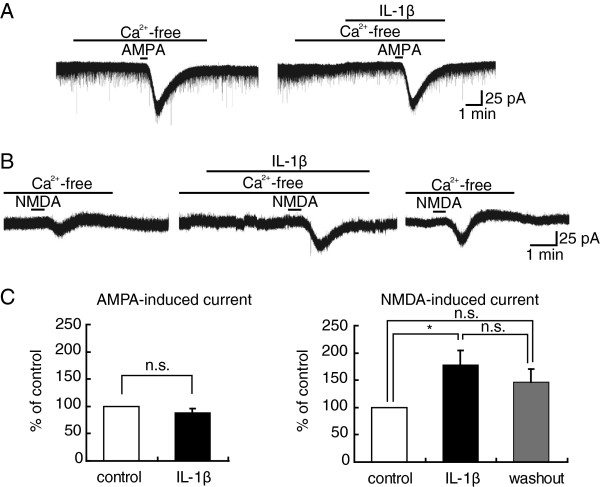
**AMPA- but not NMDA-induced current amplitude increase by IL-1β depended on extracellular Ca**^**2+**^**.** (**A**) Recordings of AMPA-induced currents in the absence and presence of IL-1β in a nominally Ca^2+^-free Krebs solution; they were obtained from the same neuron. (**B**) Recordings of NMDA-induced currents in the absence and presence of IL-1β in a nominally Ca^2+^-free Krebs solution; they were obtained from the same neuron. (**C**) Averaged peak amplitude of AMPA (left, n = 5)- or NMDA (right, n = 7)-induced currents in the presence of IL-1β (black bar), relative to those in the absence of IL-1β (control, open bar), in Ca^2+^-free solution.

Since the release of neuromodulators involving in the IL-1β effect from any neurons requires intracellular Ca^2+^, we next examined whether the potentiating effect of IL-1β was dependent on extracellular Ca^2+^. In all neurons examined, AMPA-induced currents were not increased in amplitude by IL-1β in a Ca^2+^-free bath solution (88 ± 8% (n = 5, P > 0.05) of control (68 ± 13 pA), Figure 4A). However, NMDA-induced current amplitudes were increased by IL-1β in the presence of Ca^2+^-free bath solution (178 ± 26% (n = 7, P < 0.05) of control (18 ± 5 pA), Figure 4B), and this increase was moderately enhanced 30 min after washout of IL-1β (146 ± 24% (n = 6, P > 0.05) of control (29 ± 6 pA), Figure 4B). These findings indicate that the effect of IL-1β on AMPA- but not NMDA-induced current may be due to an increase in intracellular Ca^2+^ concentration originating from extracellular Ca^2+^. Figure 4C summarizes the effects of IL-1β on AMPA- and NMDA-induced currents in Ca^2+^-free solution, which are examined in 5 and 7 neurons, respectively.

### AMPA- or NMDA-induced currents potentiated by IL-1β are modulated by glial activation

**Figure 5:**
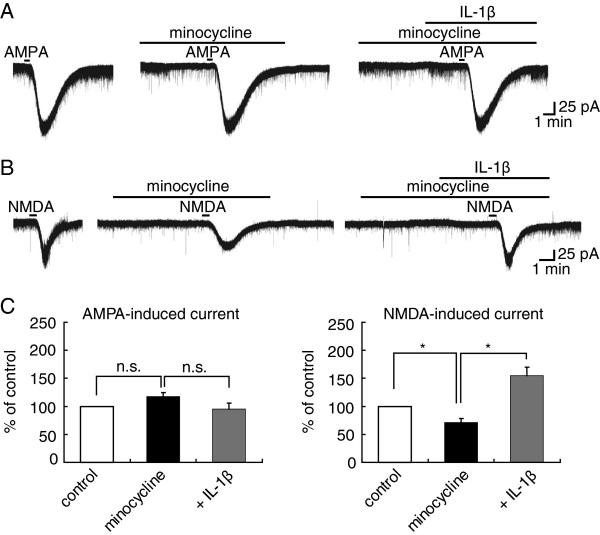
**AMPA- but not NMDA-induced current amplitude increase by IL-1β disappeared in the presence of minocycline.** (**A**, **B**) Recordings of AMPA (**A**) or NMDA (**B**)-induced currents in Krebs solution without drugs (left), with minocycline (20 μM) alone (middle) and both minocycline and IL-1β (right). In each of (**A**) and (**B**), all recordings were obtained from the same neuron. (**C**) Bar graphs showing the averaged peak amplitudes of AMPA (left, n=6)- and NMDA (right, n=5)-induced currents in the presence of IL-1β (gray bar), relative to those in the absence of IL-1β (black bar), in Krebs solution containing minocycline. Note that minocycline itself inhibited NMDA- but not AMPA-induced currents (compared to control, open bar).

Since both neuronal and glial cells are shown to express IL-1R [40], activation of IL-1R in glial cells may be implicated in persistent hyperalgesia. To know whether the modulation by IL-1β of AMPA and NMDA response is mediated by glial activation, we investigated the effect of a microglia inhibitor minocycline (20 μM) on the IL-1β mediated responses in SG neurons. As illustrated in Figure 5A and B, the potentiation of the inward current evoked by AMPA but not NMDA in the presence of IL-1β was inhibited in extent by minocycline. The percent values were, respectively, 95 ± 11% (n = 6, P > 0.05) of control (100 ± 14 pA, Figure 5A) and 155 ± 15% (n = 5, P < 0.05) of control (46 ± 17 pA, Figure 5B). The facilitatory action of IL-1β on NMDA current moderately persisted 30 min after washout of IL-1β in the presence of minocycline [174 ± 58% (n = 4, P > 0.05) of control (92 ± 30 pA), not shown]. It is important to note that minocycline *per se* inhibited the NMDA- (71 ± 7% (n = 6, P < 0.05) of control (63 ± 19 pA), Figure 5B) but not AMPA-induced current (117 ± 8% (n =5, P > 0.05) of control (82 ± 16 pA), Figure 5A). These results suggest that glial cells located in the spinal dorsal horn play an important role in pain transmission conveyed by IL-1β. Figure 5C summarizes the effects of IL-1β on AMPA- and NMDA-induced currents in the presence of minocycline, which are examined in 6 and 5 neurons, respectively. These results together indicate that microglia activation in the spinal dorsal horn may be involved in the IL-1β induced sensitization of SG neurons.

## Discussion

In the present study, we investigated the effect of IL-1β on AMPA- and NMDA-induced currents in SG neurons by using whole-cell patch-clamp recordings. Our study shows the enhancement by IL-1β of both postsynaptic AMPA and NMDA receptor responses which has been reported previously [39]. Moreover, our data reveal that these facilitatory effects of IL-1β on AMPA and NMDA receptors may reflect different mechanisms. Importantly, this is the first study to demonstrate that pretreatment with IL-1ra blocks the IL-1β-mediated potentiation of responses to exogenously-applied AMPA or NMDA. Our data further provide new and important evidence that microglia activation may be involved in the glutamate-receptor response potentiations produced by IL-1β.

The role of ionotropic glutamate receptors (mainly AMPA and NMDA receptors in SG neurons) as targets of analgesics has emerged during the last decade. It has been reported that the activation of NMDA receptors critically contributes to the development of chronic nociceptive hypersentivity following peripheral tissue damage or nerve injuries [41]. Recently, some studies have also shown a contribution of spinal AMPA receptors in the development of both acute and chronic painful conditions [42,43]. There is still an ongoing controversy whether IL-1β signaling is involved in pain transmission under normal, non-inflammatory states. When injected intrathecally into intact rats, IL-1β is reported to be without effect [20,44]. However, our study clearly demonstrates that acute application of IL-1β rapidly alters the efficacy of extrasynaptic AMPA and NMDA receptors in SG neurons of spinal cord slices. This may explain the fact that intrathecal administration of IL-1β in healthy rats induces hyperalgesia and allodynia and enhances wind-up activity in dorsal horn neurons [45]. Recently, several groups of SG neurons with distinct electrophysiological properties were characterized [46,47]. Although we cannot be sure that the excitatory actions of IL-1β in our study are exerted on excitatory interneurons, it is generally believed that most neurons recorded in the SG are excitatory [48,49]. In addition, IL-1β has been reported to depolarize paraventricular nucleus neurons [32] or to hyperpolarize hypothalamic neurons [33], but this was not the case in SG neurons, because currents required to hold membrane potentials at −70 or −50 mV were unaffected by IL-1β. In accordance with data showing that IL-1β increases NMDA receptor-mediated rise in intracellular calcium levels in hippocampal neurons [50] and enhances NMDA-induced current in cultured hippocampal [51] or SG neurons [39], our data also demonstrated an enhancement of NMDA current by IL-1β. Although it was reported that AMPA-induced current was moderately enhanced by IL-1β (P > 0.05) [39], our data demonstrated that it was significantly enhanced by IL-1β. This discrepancy may be due to a longer superfusion time of IL-1β in our studies and indicate that the effects of IL-1β on neurons depend on not only the type of neurons examined but also the duration of time that the neuron is exposed to it. In particular, our results confirm that IL-1β exhibited a persistent facilitatory effect on postsynaptic NMDA but not AMPA receptor function after removal of this cytokine. Thus, IL-1β may activate a postsynaptic process linked to long-term potentiation (LTP) through NMDA receptors which might have functional relevance for hyperalgesia or allodynia. This supports the report that IL-1β produced a thermal hyperalgesia lasting for 24 h after its intrathecal injection [26].

IL-1RI, which is known to mediate all biological functions of IL-1β [52], was detected in most of large and small DRG neurons as well as in some glia-like cell types by *in situ* hybridization staining [53]. Another double immunofluorescence labeling study showed that IL-1RI and NMDA receptor NR1 subunit are co-localized in the spinal dorsal horn [29]. Consistently, acute or chronic administration of IL-1ra, the endogenous antagonist of the IL-1R, which competitively blocks the binding of IL-1β to the receptor without inducing a signal of its own [54], inhibits a hypernociception induced by IL-1β [16,19,55-57]. Thus, inhibition of IL-1β signaling such as blockade of IL-1RI by IL-1ra in spinal dorsal horn neurons could account for its antinociceptive action and decrease the function of excitatory transmission in pain pathway. In agreement with these reports, our present data demonstrate that IL-1ra inhibits AMPA- and NMDA-induced current increases produced by IL-1β in all the neurons tested. Based on reports that NMDA receptors have an essential role in pain hypersensitization, our findings provide new and important evidence that inhibition by IL-1ra of NMDA receptor potentiation produced by IL-1β could prevent plasticity-related phenomena such as central sensitization or excitotoxicity. Actually, IL-1ra prevents the development of LTP, presumably by inhibiting NMDA receptor phosphorylation. An immunohistochemical study demonstrated that IL-1ra inhibited spinal cord phosphorylation of NR1 in a rat model of inflammatory pain [29]. In another study, IL-1ra abolished NMDA-induced intracellular Ca^2+^ level increases produced by IL-1β, which may be involved in preventing tyrosine phosphorylation of NMDA receptor NR2A/B subunit [50]. Compared to cultured or acutely-isolated neurons, neurons in spinal cord slices used in the present study have the advantage of offering recording under conditions closer to physiological ones. Our electrophysiological experiment data provide a cellular mechanism that IL-1RI may be a target for treatment of inflammatory and neuropathic pain.

Several cellular mechanisms could be involved in the enhancement of postsynaptic AMPA and NMDA receptor responses by IL-1β, e.g., presynaptic transmitter release, the modulation of the receptors in quantity (*via* a change in trafficking) and efficacy. It has been observed that IL-1β could increase the release of glutamate through an increase in Ca^2+^ influx in hippocampal neurons [58]. The present study also demonstrates that the facilitatory effect of IL-1β on postsynatpic AMPA receptors was TTX-sensitive and Ca^2+^-dependent since AMPA-induced current increased by IL-1β was abolished in the presence of TTX or Ca^2+^-free Krebs solution. Consistent with the report that IL-1β increased the frequency and amplitude of spontaneous excitatory postsynaptic currents (sEPSCs) in SG neurons [39], our data indicated that the enhancement of AMPA responses may be generated by glutamate-receptor activation and neuronal activity increase, possibly by the facilitatory effect of IL-1β on intracellular Ca^2+^ level elevation. On the other hand, NMDA receptor response facilitation produced by IL-1β was resistant to TTX or Ca^2+^-free, indicating a direct action on postsynaptic NMDA receptor. The glutamate-mediated excitatory transmission efficiency is dependent on the number and function of AMPA or NMDA receptors at glutamatergic synapses. It is difficult from our data to know postsynaptic AMPA or NMDA receptor regulation such as receptor trafficking or subunit phosphorylation by IL-1β, but our findings are consistent with previous studies that the former is probably associated with a presynaptic mechanism of glutamate release increase while the latter is mediated by NMDA receptor subunit phosphorylation.

IL-1β is a cytokine released from spinal glial cells in response to pathophysiological changes that occur during different disease states, such as inflammatory and neuropathic pain. Initial reports suggested that IL-1β is an extremely potent hyperalgesic agent when injected systemically, intraperitoneally or intraplantarly in rats [14]. These results may be in accordance with literatures reporting that activation of microglia and astrocyte may be involved in neuropathic pain [59,60] and strongly suggest signaling *via* IL-1β between neuronal and glial cells might occur. Our results showed that in the presence of a microglia inhibitor minocycline, AMPA-induced current amplitude increase mediated by IL-1β was inhibited without any changes by minocycline itself. In contrast, NMDA-induced current facilitation produced by IL-1β was not affected by minocycline. Minocycline-sensitive microglia may be activated by neurotransmitters released as a result of an increase in neuronal activity, resulting in an enhancement of AMPA but not NMDA response. This issue remains to be further addressed. Furthermore, we reported, for the first time, that minocycline *per se* depressed NMDA-induced current. This inhibitory action may be due to an open-channel block by minocycline itself, as shown for GluR2 homomeric channels [61]. Further research is needed to elucidate the molecular mechanism underlying this NMDA receptor inhibition by minocycline in SG neurons. Consistent with others’ data, our study indicates the realization that glia-derived signaling molecules such as IL-1β can contribute to and modulate pain signaling in the spinal cord.

## Conclusions

In summary, we demonstrate novel glia-neuron mechanisms by which acute application of IL-1β directly enhances AMPA- and NMDA-receptor responses in a subpopulation of SG neurons in rat spinal cord slice preparations, effects that are a result of the activation of IL-1RI by this cytokine and also of microglial cells. Specifically, the potentiation by IL-1β of postsynaptic NMDA but not AMPA receptors persisted. This distinct plastic change suggests that IL-1β exerts a crucial role in not only acute but also pathological pain sensation. IL-1ra and microglia inhibitors may be useful candidates for the treatment of pain.

## Methods

All animal experiments were approved by the Animal Care and Use Committee of Saga University (Saga, Japan) and were conducted in accordance with the UK Animals (Scientific Procedures) Act of 1986 and associated guidelines.

### Spinal cord slice preparation

The methods used to obtain adult rat spinal cord slices were similar to those used in our previous studies [62-65]. In brief, male adult Sprague–Dawley rats (6–8 wk old, 200–300 g) were anesthetized with urethane (1.2 g/kg, intraperitoneal), and then a laminectomy was performed to extract a lumbosacral spinal cord enlargement (L1–S3). The spinal cord was removed and placed in preoxygenated ice-cold (1–3°C) Krebs solution. Immediately after the removal of the spinal cord, the rats were given an overdose of urethane and then sacrificed by exsanguination. The pia-arachnoid membrane was removed after cutting all the ventral and dorsal roots near the root entry zone. The spinal cord was mounted on a vibrating microslicer (DTK-1000; Dousaka, Kyoto, Japan), and several 600-μm-thick transverse slices were cut. One of the slices was placed on a nylon mesh in the recording chamber (volume: 0.5 ml) and then perfused at a rate of 10–15 ml/min with Krebs solution saturated with 95% O_2_ and 5% CO_2_, and maintained at 36 ± 1°C. The composition of the Krebs solution used was as follows (in mM): 117 NaCl, 3.6 KCl, 2.5 CaCl_2_, 1.2 MgCl_2_, 1.2 NaH_2_PO_4_, 25 NaHCO_3_, and 11 glucose (pH = 7.4 when saturated with the gas). The remaining slices were stored under similar conditions until use. Typically, one neuron was studied per slice to observe the effect of IL-1β.

### Patch-clamp recordings from SG neurons

The SG was identified as a translucent band under a binocular microscope with light transmitted from below [62-64]. Blind whole-cell patch-clamp recordings were performed from neurons located at the center of SG to avoid recordings from the laminae I and III neurons. Patch pipettes were fabricated from thin-walled, fiber-filled capillaries (1.5 mm OD, World Precision Instruments, USA) and contained the following solutions (in mM): 135 K-gluconate, 5 KCl, 0.5 CaCl_2_, 2 MgCl_2_, 5 EGTA, 5 HEPES, and 5 Mg-ATP (pH = 7.2). The patch pipettes had a resistance of 8–12 MΩ. Signals were acquired using an Axopatch 200B amplifier (Molecular Devices, Sunnyvale, CA, USA). Currents obtained in the voltage-clamp mode were low pass filtered at 5 kHz and digitized at 333 kHz with an analog-to-digital converter (Digidata 1322A, Molecular Devices). Holding potential was set to −70 mV for recording exogenous AMPA current. Exogenous NMDA current was recorded at −50 mV. The data were stored and analyzed with a personal computer using pCLAMP v 9.2 software (Molecular Devices).

### Application of drugs

Drugs were applied by perfusion *via* a three-way stopcock without an alteration in the perfusion rate or temperature. The solution in the recording chamber having a volume of 0.5 ml was completely replaced within 15 s. The drugs used were IL-1β (rat recombinant), IL-1ra (human, Cedarlane Laboratories Ltd, ON, Canada), AMPA (Tocris Cookson, Bristol, UK), NMDA, minocycline hydrochloride (Sigma-Aldrich, St. Louis, MO, USA) and TTX (Wako, Osaka, Japan). These drugs (except for IL-1β and IL-1ra) were first dissolved in distilled water at 1,000 times the concentration to be used and then stored at −20°C. 1000x stock solutions of IL-1β and IL-1ra were prepared in phosphate buffered saline (PBS) containing 1% bovine serum albumin (BSA) (Sigma-Aldrich) and stored at −20°C. The stock solution was diluted to the desired concentration in Krebs solution immediately before use. To prevent a desensitization of AMPA and NMDA receptor during bath application of agonist to slices, time intervals between the repetitive application of AMPA and NMDA were > 30 min. The tubing of our perfusion system was coated with AquaSil siliconizing fluid (Thermo Fisher Scientific, USA) to reduce the loss of IL-1β and IL-1ra through nonspecific interactions. The tonicity of the nominally Ca^2+^-free, high-Mg^2+^ (5 mM) Krebs solution was adjusted by lowering the Na^+^ concentration of the Krebs solution.

### Statistical analysis

Numerical data are presented as the means ± SEM. Statistical significance was determined as *P* < 0.05 using the Student's paired *t*-test to compare groups treated or non-treated with IL-1β. In all cases, *n* refers to the number of neurons recorded.

## Competing interests

The authors declare that they have no competing interests.

## Authors’ contributions

TL conceived and designed the study, carried out the patch-clamp recordings, performed the statistical analysis and wrote the manuscript. CHJ participated in the patch-clamp recordings. TF participated in the patch-clamp recordings. SWL participated in the study design. EK participated in the study design and reviewed the manuscript. All authors read and approved the manuscript. 
